# Alcohol dehydrogenase system acts as the sole pathway for methanol oxidation in *Desulfofundulus kuznetsovii* strain TPOSR

**DOI:** 10.1007/s10482-024-01937-1

**Published:** 2024-03-01

**Authors:** Lukas Friedeheim, Sjef Boeren, Irene Sánchez-Andrea, Alfons J. M. Stams, Diana Z. Sousa

**Affiliations:** 1 Laboratory of Microbiology, Wageningen University and Research, Wageningen, The Netherlands; 2 Laboratory of Biochemistry, Wageningen University and Research, Wageningen, The Netherlands

**Keywords:** Alcohol metabolism, Alcohol dehydrogenase, Methanol methyltransferase, Sulphate-reducing microorganisms

## Abstract

*Desulfofundulus kuznetsovii* is a thermophilic, spore-forming sulphate-reducing bacterium in the family *Peptococcaceae*. In this study, we describe a newly isolated strain of *D. kuznetsovii*, strain TPOSR, and compare its metabolism to the type strain *D. kuznetsovii* 17^T^. Both strains grow on a large variety of alcohols, such as methanol, ethanol and propane-diols, coupled to the reduction of sulphate. Strain 17^T^ metabolizes methanol via two routes, one involving a cobalt-dependent methyl transferase and the other using a cobalt-independent alcohol dehydrogenase. However, strain TPOSR, which shares 97% average nucleotide identity with *D. kuznetsovii* strain 17^T^, lacks several genes from the methyl transferase operon found in strain 17^T^. The gene encoding the catalytically active methyl transferase subunit B is missing, indicating that strain TPOSR utilizes the alcohol dehydrogenase pathway exclusively. Both strains grew with methanol during cobalt starvation, but growth was impaired. Strain 17^T^ was more sensitive to cobalt deficiency, due to the repression of its methyl transferase system. Our findings shed light on the metabolic diversity of *D. kuznetsovii* and their metabolic differences of encoding one or two routes for the conversion of methanol.

**Supplementary Information:**

The online version contains supplementary material available at 10.1007/s10482-024-01937-1.

## Introduction

*Desulfofundulus* is a genus of strictly anaerobic, sulphate-reducing bacteria that was reclassified from the genus *Desulfotomaculum* (Watanabe et al. 2018). *Desulfofundulus* species are thermophilic, spore-forming and stain Gram-negative, despite commonly expressing a Gram-positive resembling cell wall structure (Visser et al. 2014). Due to the ability to produce endospores and to their versatile metabolism, *Desulfofundulus* species can survive in a diversity of environments, including extreme ones. For example, *Desulfofundulus salinum* 435^T^ (formerly *Desulfotomaculum salinum*) was isolated from condensate water of the Igrim high-temperature gas field (Nazina et al. 2005), *Desulfofundulus luciae* SL^T^ (formerly *Desulfotomaculum luciae*) was isolated from a hot spring, and *Desulfofundulus kuznetsovii* 17^T^ (formerly *Desulfotomaculum kuznetsovii*) was isolated from thermal groundwater (Liu et al. 1997; Nazina et al. 1988). *D. kuznetsovii* 17^T^ is the type strain of the genus *Desulfofundulus* (Watanabe et al. 2018). It utilizes a wide range of substrates including organic acids, such as acetate, lactate, pyruvate, fumarate and succinate as well as several alcohols such as methanol, ethanol and propanol (Nazina et al. 1988). Degradation of these substrates can be coupled to reduction of sulphate or sulphite to sulphide, and production of acetate and/or CO_2_. It can also grow autotrophically with H_2_/CO_2_ and sulphate.

**Fig. 1:**
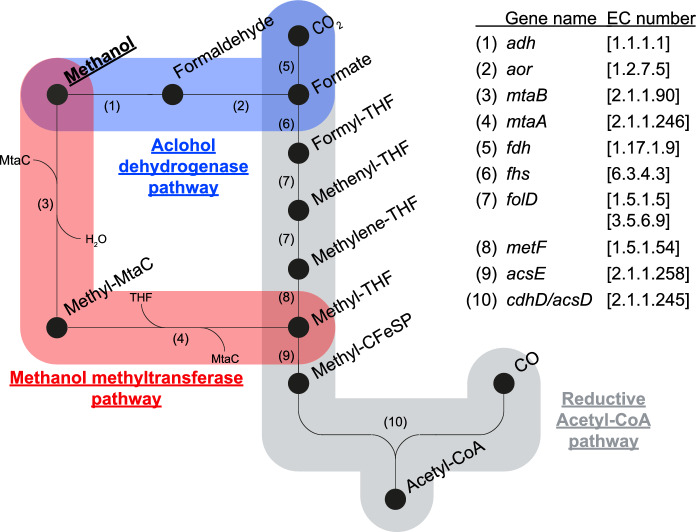
Methanol metabolism of *Desulfofundulus kuznetsovii* 17^T^. Co-occurring ADH and MT pathway are depicted in blue and red, respectively. Integration into the reductive acetyl-CoA pathway in grey is indicated. Numbers represent the enzymes catalysing each the reactions, with corresponding gene names and EC numbers are given on the side of the image

Recent research by Sousa et al. (2018) has elucidated the methanol metabolism in *D. kuznetsovii* 17^T^, revealing the co-occurrence of two methanol utilization pathways, a cobalt-dependent methyl transferase (MT) pathway and a cobalt-independent, NAD-dependent alcohol dehydrogenase (ADH) pathway as depicted in Fig. 1. Typically, aerobic and facultative anaerobic microorganisms utilize ADHs (EC:1.1.1.1) and aldehyde ferredoxin oxidoreductases (AORs, EC:1.2.7.5) to oxidize methanol to formaldehyde and formate, while anaerobic organisms such as acetogenic bacteria and methanogenic archaea use methanol methyltransferase (MT) systems instead. The MT systems consist of three main subunits that play a role in methanol metabolism. Subunit B (MtaB, EC:2.1.1.90) catalyses the cleavage of the hydroxyl bond in methanol and transfers the resulting methyl group to the subunit C (MtaC). This step requires a cobalamin cofactor with reduced Co(I), making this pathway highly dependent on cobalt availability and inhibited by oxygen. Subsequently, subunit A (MtaA, EC:2.1.1.246) catalyses the transfer of the methyl group from MtaC to coenzyme M in methanogens (Harms and Thauer 1996; van der Meijden et al. 1983) or to tetrahydrofolate in acetogens (Das et al. 2007; Stupperich and Konle 1993; Zhou et al. 2005). In the study by Sousa et al. (2018), stable isotope fractionation indicated that during growth of *D. kuznetsovii* 17^T^ with methanol the ADH is used first and the MT is employed later at lower methanol concentrations. This discovery of two possible methanol conversion pathways in the same microorganism raises questions about the prevalence of the two-pathway system in sulphate-reducing bacteria and its impact on physiological performance in environments such as deep sub-surfaces.

In this work we try to answer some of these questions by means of genomic and proteomic comparison of *D. kuznetsovii 17*^*T*^ to a novel isolated strain of the *D. kuznetsovii* species, strain TPOSR [Digital DNA-DNA hybridization: 80.6% with species cutoff at dDDH < 70% (Auch et al. 2010)]. Despite their high genome identity and similar physiological characteristics, strain TPOSR lacks the essential genes of the MT system rendering the strain reliant on ADH for methanol metabolism. Interestingly, regardless of its reliance on the ADH pathway the most abundant ADH during growth with methanol is virtually identical to the one expressed in strain 17^T^ with its two-pathway system. In physiological studies with methanol, cobalt starvation impaired growth of both strains, with strain 17^T^ being more affected likely due to MT suppression.

## Materials and methods

### Enrichment and isolation of *Desulfofundulus kuznetsovii* strain TPOSR

Strain TPOSR was isolated from sludge withdrawn from an anaerobic blanket (UASB) reactor operated at 55 °C with propionate as sole carbon and energy source. The sludge sample for microbial enrichments was obtained from the reactor’s bulk medium without prior homogenization of the sludge material. Microbial enrichments were carried out with propionate (10 mM) and sulphate (20 mM) using basal medium prepared as described below; bromoethane sulfonic acid was added to the medium to a final concentration of 20 mM to prevent growth of methanogens relying on methyl transferase systems. Resulting culture was transferred to a rich medium containing acetate (10 mM), propionate (10 mM), ethanol (10 mM), glucose (10 mM) and yeast extract (1%), with a gas phase of H_2_/CO_2_ (80/20%, 1.7 atm), with and without sulphate (20 mM) as electron acceptor. Growth was just observed in the presence of sulphate. A pure culture of strain TPOSR was obtained by serial dilution. Purity of the culture during this study was routinely verified by microscopy using a Leica DM2000 microscope (Leica, Microsystems, Weltzar, Germany) and 16S rRNA gene Sanger sequencing [using universal bacteria primers 27F and 1492RV (Lane et al. 1985)]. Strain TPOSR was deposited at the German Collection of Microorganisms and Cell Cultures (DSMZ; Braunschweig, Lower Saxony, Germany) under the accession number DSM 110707.

### Microorganisms and cultivation

All cultivations were carried out in serum bottles filled with basal anoxic bicarbonate-buffered medium (Stams et al. 1993). Bottles were sealed with rubber caps and aluminium cramps, and the headspace was flushed and pressurized with N_2_/CO_2_ [80/20% (v v^−1^), 1.7 atm] prior to autoclavation. Vitamins, electron donors and acceptor were added after autoclavation to the medium from separately sterilized anaerobic stock solutions. Electron donors and acceptors were supplemented if applicable to the medium at a final concentration of 5 mM and 20 mM, respectively, unless mentioned otherwise. Bottles were inoculated with 1% (v v^−1^) inoculum from exponentially growing cultures of strains 17^T^ or TPOSR. Cultures were incubated statically at 55 °C in the dark. All used chemicals were procured from Sigma-Aldrich (St. Louis, MO).

### Physiological characterization of strain TPOSR

Cell morphology was assessed by phase contrast microscopy using a Leica DM2000 microscope (Leica, Microsystems, Weltzar, Germany). Utilization of the following electron donors was tested in presence of 20 mM sulphate: organic acids: formate (20 mM), acetate (10 mM), glycolate (5 mM), pyruvate (5 mM), propionate (5 mM), lactate (5 mM), malate (5 mM), fumarate (5 mM), butyrate (5 mM), succinate (5 mM), citrate (5 mM), benzoate (5 mM); alcohols: methanol (20 mM), ethanol (10 mM), ethylene–glycol (5 mM), 1-propanol (5 mM), 2-propanol (5 mM), 1,2-propanediol (5 mM), 1,3-propanediol (5 mM), glycerol (5 mM); amino acids (5 mM): cysteine, serine, isoleucine, leucine, alanine, arginine, and sugars (5 mM): xylose, fructose, glucose, maltose, sucrose, starch. Growth on H_2_/CO_2_ (80/20% v v^−1^ at 1.6 bar) and CO/CO_2_ (20/80% v v^−1^ at 1.6 bar) was tested by exchanging the headspace to the designated gas mix in medium containing 20 mM sulphate or without sulphate. The following electron acceptors (10 mM) were tested, with methanol (20 mM) as electron donor: sulphate, sulphite, thiosulphate, elemental sulphur, fumarate, nitrate and nitrite (2 mM). Temperature, pH, and salinity optima were determined in media with methanol (20 mM) and sulphate (20 mM). Temperature range for growth was determined in the range 30–90 °C in intervals of 5 °C. Effect of NaCl concentration was evaluated, in addition to the 0.3 g l^−1^ of the basal medium, in a range of 0–3% (w v^−1^) in 0.5% steps. For determination of optimal pH, unbuffered media was adjusted with KOH and HCl to a range of 5.5–9 (5.5, 6.1, 6.5, 6.9, 7.3, 7.9, 8.1, 8.4, 9.0).

### Genome sequencing, assembly, and annotation

Strain TPOSR was grown anaerobically at 60 °C in bicarbonate-buffered basal medium supplemented with methanol (20 mM) and sulphate (20 mM). Genomic DNA was extracted from 100 ml of exponentially grown cultures, pelleted at 5000×*g* for 15 min using the Lucigen MasterPure DNA purification kit (ThermoFisher, Waltham, MA), according to manufacturer’s instructions. DNA quality was checked using 1.5% agarose gel electrophoresis and spectrophotometric quantification with a DS-11 series Spectrophotometer (DeNovix, Wilmington, DE). DNA was sequenced at Novogene (Cambridge, United Kingdom) with a 50 × Pacbio sequel system, resulting in 180,000 reads with an average length of 4 kb and a N50 value of 55%. The reads were assembled using flye version 2.6 (Kolmogorov et al. 2019) resulting in the generation of 5 contigs with a total length of about 3.7 mb. Quality of the assembly was evaluated using CheckM version 1.0.18 (Parks et al. 2015) and Quast version 4.4 (Gurevich et al. 2013), with an estimated genome completeness of 99.2% with 1.9% contamination. Annotation was carried out following the NCBI prokaryotic genome annotation pipeline version 4.11 (Tatusova et al. 2016). A table of the protein IDs used in this study for both strain TPOSR and strain 17^T^ with corresponding reference sequences and locus tags can be found in the Online Resource 1. Data is deposited under NCBI accession NZ_JAAOEF000000000 (BioProject: PRJNA224116, BioSample: SAMN14245301, Assembly: GCF_011393055.1).

### Phylogenetic analysis

For whole genome based taxonomic analysis the Type (Strain) Genome Server (TYGS, https://tygs.dsmz.de) was used (Meier-Kolthoff et al. 2021; Meier-Kolthoff & Göker 2019). The results were obtained from the TYGS on 07.03.2023. For the phylogenomic inference, all pairwise comparisons among the set of genomes were conducted using GBDP and accurate intergenomic distances inferred under the algorithm 'trimming' and distance formula d5 (Meier-Kolthoff et al. 2013). 100 distance replicates were calculated each. Digital DDH values, average nucleotide identity (ANI) and confidence intervals were calculated using the recommended settings of the GGDC 3.0 (Meier-Kolthoff et al. 2021), using reference genomes for comparison. The resulting intergenomic distances were used to infer a balanced minimum evolution tree with branch support via FASTME 2.1.6.1 including SPR postprocessing (Lefort et al. 2015). Branch support was inferred from 100 pseudo-bootstrap replicates each. The trees were rooted at the midpoint (Farris 1972).

### Comparative proteomics

Strain TPOSR was grown in media with methanol (20 mM) and sulphate (20 mM), in both presence and absence of cobalt and vitamin B12. As a non-alcohol control condition for proteomics studies, we analysed the proteome of *D. kuznetsovii* TPOSR cells grown with lactate (20 mM) and sulphate (20 mM). Assays were done in triplicate. For protein extraction, 50 ml of mid-exponential phase grown cells were harvested by centrifugation at 10,000*g* at 4 °C for 10 min. Pelleted cells were washed and subsequently resuspended in 100 mM Tris–HCl (pH 8). To disrupt cells, sonication was performed using a sonicator equipped with a MS72 microtip probe (Bandelin, Germany) at 50% amplitude for 15 s. Cell debris was removed by centrifugation at 10,000*g* at 4 °C, and the supernatant was reduced using 15 mM DTT for 30 min at 45 °C. Proteins were denatured using 6 M urea, alkylated with 20 mM acrylamide (incubated at 21 °C for 30 min). The pH was adjusted to 7 using 10% trifluoroacetic acid (TFA). Protein aggregation capture as used to capture proteins by using 1 μm diameter magnetic carboxylate modified beads (GE Healthcare, Brøndby, Denmark) (Batth et al. 2019). 100% Acetonitrile (2 times current volume) was added to induce protein aggregation during 20 min shaking at room temperature. Beads were retained on the wall of the tube using magnets and washed with 70% ethanol in water and 100% acetonitrile. Remaining protein was digested overnight at room temperature by addition of 100 µl ammonium bicarbonate (50 mM) containing 0.5 µg sequencing grade trypsin. The digested peptides were acidified to pH 3 using 10% TFA. Subsequently, the beads were retained and the solution was filtered through a double layer C8 extraction disk (CDS analytical, Oxford, PA). Filter trapped peptides were eluted with a 50% acetonitrile and 0.1% formic acid solution and concentrated by evaporation of the organic solvent. Protein concentration was measured using the Pierce Bradford Protein Assay Kit (ThermoFisher, Waltham, MA).

Per sample, 0.5 µg of protein was loaded directly onto a 0.10 * 250 mm ReproSil-Pur 120 C18-AQ 1.9 µm beads (Dr. Maisch, Germany, Ammerbuch-Entringen) analytical column (prepared in-house) at a constant pressure of 825 bar (flow rate of circa 600 nl min^−1^) with buffer (1% formic acid in water) and eluted at a flow of 0.5 µl min^−1^ with a 50 min linear gradient from 9 to 34% acetonitrile in water with 1% formic acid with a Thermo EASY nanoLC1000 (ThermoFisher, Waltham, MA). An electrospray potential of 3.5 kV was applied directly to the eluent via a stainless-steel needle fitted into the waste line of a micro cross that was connected between the nLC and the analytical column. On the connected Orbitrap Exploris 480 (ThermoFisher, Waltham, MA) MS and MSMS AGC targets were set to 300%, 100% respectively or maximum ion injection times of 50 ms (MS) and 30 ms (MSMS) were used. HCD fragmented (isolation width 1.2 *mz*^−1^, 28% normalized collision energy) MSMS scans in a cycle time of 1.1 s the most abundant 2–5 + charged peaks in the MS scan were recorded in data dependent mode (Resolution 15,000, threshold 2e4, 15 s exclusion duration for the selected *mz*^−1^ ± 10 ppm). LC–MS/MS runs with all MS/MS spectra obtained were analysed with MaxQuant 1.6.3.4 (Cox et al. 2014). Peptides and proteins were considered reliable for further analysis when they had a false discovery rate (FDR) < 1% and when of a protein at least two peptides were identified of which at least one was unique and one unmodified. Data analysis was performed using Perseus version 1.6.2.1 (Tyanova et al. 2016). Abundance values were log normalized with respect to the total amount of protein and the identified peptides of each. The relative protein abundances were represented as Log10-transformed LFQ values. Student’s *t* test was performed using the “LFQ intensity” columns obtained with a significance level of *p* ≤ 0.05 and S0 = 0.1. Fold change was expressed as the ratio of averaged LFQ value of a protein across all replications between the two compared conditions. The mass spectrometry proteomics data have been deposited to the ProteomeXchange Consortium via the PRIDE partner repository with the dataset identifier PXD047013 (Perez-Riverol et al. 2022).

### Physiological comparison of growth with methanol with and without cobalt and vitamin B12

To assess the cobalt dependency of growth with methanol, both strains were grown in bottles containing methanol and sulphate, with and without cobalt and vitamin B12. Growth conditions were as mentioned above but cobalt-free medium was prepared by omitting the addition of cobalt and vitamin B12 from the vitamin and trace element solutions described by Stams et al. (1993). Addition of vitamin B12 was done in form of cyanocobalamin. Preventatively, bottles used for cobalt-free condition were cleaned with acidic solution (aqua regia—HNO_3_ and HCl 1/3 mol mol^−1^) to remove any lingering cobalt and other residues. All cultures grown in cobalt-free medium were adapted to this condition for several transfers beforehand (minimum 10 transfers in cobalt-free condition).

### Analytical techniques

Microbial growth was tracked by measuring absorption at OD_600_ measured in a UV-1800 UV–Vis spectrophotometer (Shimadzu, Japan). Methanol concentration of the media was measured using a GC-2010 (Shimadzu, Japan) equipped with a DB-WAX UI column (Agilent, Santa Clara, CA) with N_2_ as carrier gas (at a constant pressure of 100 kPa) and using a flame ionization detector (FID) (at a temperature of 250 °C). Samples were injected using a headspace auto sampler HS-20 (Shimadzu, Japan) with an oven set to 50 °C.

## Results and discussion

### Characterization of strain TPOSR

Strain TPOSR was isolated from sludge collected from an anaerobic digester fed with propionate. Cells of strain TPOSR were straight to slightly curved rods, 0.5–0.8 µm wide and 2–5 µm long after a growth period of 10 days with methanol (20 mM). Cells stained Gram-negative and were non-motile. Spores were observed central, leading to central swelling of the cell. Substrate depletion caused cells to form chains of longer than two units, and it induced spore formation. The temperature range for growth of strain TPOSR was 50–70 °C with an optimum at 55–60 °C. Growth was observed at pH values between 6.5 and 7.9, with optimal growth at pH 7.3. Strain TPOSR exhibited tolerance to NaCl concentrations up to 1.5%, with optimal growth observed without extra NaCl added over the basal 0.3 g l^−1^ concentration of the medium. Yeast extract slightly increased the growth rate but was not required for growth (and therefore omitted in further tests). With sulphate as electron acceptor, strain TPSOR grew on the following substrates: formate, acetate, pyruvate, propionate, lactate, malate, fumarate, butyrate, succinate, methanol, ethanol, ethylene glycol, 1-propanol, 1–2-propanediol, 1–3 propanediol and butanol. Autotrophic growth on H_2_/CO_2_ and CO was observed with sulphate as electron acceptor but not without. Acetate was oxidized completely to CO_2_ and genes involved in the acetyl-CoA pathway were found in the genome of strain TPOSR. Organic acids—propionate, pyruvate and butyrate—are mostly oxidized completely to CO_2_, with small amounts of intermediate acetate formation. Growth on alcohols—propanol and butanol—yielded first the formation of the corresponding acid and subsequent conversion to acetate. Pyruvate was fermented to acetate, lactate to acetate and propionate, and fumarate was fermented to acetate, propionate and traces of malate. No growth was observed on sugars or amino acids. The strain reduced sulphite and thiosulphate, in addition to sulphate. Elemental sulphur, nitrate, nitrite and fumarate were not used as electron acceptors (Table 1).

**Table 1:** Characteristics of strain TPOSR compared with its closest relatives in the genus *Desulfofundulus*: *D. kuznetsovii* (Nazina et al. 1988), *D. thermosubterraneus* (Kaksonen et al. 2006), *D. salinum* (Nazina et al. 2005), *D. australicus* (Love et al. 1993) and *D. thermocisternus* (Nilsen et al. 1996)

	*D. TPOSR*	*D. kuznetsovii*	*D. thermosubterraneus*	*D. salinum*	*D. australicus*	*D. thermocisternus*
Type strain	TPOSR	17	RL50JIII	435	AB33	ST90
Morphology	Single or in pairs/rod	Single or in pairs/rod	Single or in pairs/rod		Single or in pairs/rod	Single or in pairs/rod
Isolation source	Methanogenic granular sludge from SBR	Thermal mineral water	Geothermal mine in Japan	Oil strata	Nonvolcanically heated Great Artesian Waters	North Sea oil reservoir formation water
Cell size (μm)	2–5 * 0.5–0.8	1–1.4 * 3.5–5	0.8–1.0 * 3–10	0.9–1.3 * 2.5	0.8–1 * 3–6	0.7–1 * 2–5.2
Endospore position	Central	Central	Central/subterminal	Terminal	Central/terminal	Central
Motility	+	+	+	+	+	+
Gram staining	Neg. but positive cell wall structure	Neg. but positive cell wall structure	Positive	Neg. but positive cell wall structure	Neg. but positive cell wall structure	Positive cell wall structure
Temp. range (°C)	50–65	50–85	50–72	40–70	40–74	41–75
Temp. optimum (°C)	60	60–65	61–66	60–65	68	62
pH range	6.5–7.9	4.15–9.9	6.4–7.8	6–8.5	5.5–8.5	6.2–8.9
pH optimum	7.3	7–7.2	7.2–7.4	7	7–7.4	6.7
NaCl range (%)	0–1.5	0–3	0–1.5	0–6	> 0	0–4.5
NaCl optimum (%)	0	0	0–1	0.5–1	NR	0.3–1
DNA G + C mol%	55.2	54.9	54.5	55.1	54.6	54.4
Genome size (Mb)	3.76	3.6	3.41	2.89	2.88	2.9
*Electron donors with sulphate*						
H_2_/CO_2_/sulphate	+	+	+	+	+	+
Formate	+	+	+	+	−	−
Acetate	+	+	−	(+)	+	−
Glycolate	−	NR	NR	NR	NR	NR
Pyruvate	+	+	+	+	+	+
Propionate	+	+	+	NR	−	+
Lactate	+	+	NR	+	+	+
Malate	+	+	+	+	NR	NR
Fumarate	+	+	+	+	−	NR
Butyrate	+	+	+	+	−	+
Succinate	+	+	+	+	−	NR
Citrate	−	−	NR	NR	NR	NR
Benzoate	−	−	−	NR	+	−
Methanol	+	+	−	+	−/+*	−
Ethanol	+	+	+	+	+	+
Ethylene glycol	+	NR	NR	NR	NR	NR
1-Propanol	+	+	+	+	NR	+
2-Propanol	−	−	NR	NR	NR	NR
1–2-Propane-diol	+	+*	NR	NR	NR	NR
1–3-Propane-diol	+	+*	NR	NR	NR	NR
Glycerol	−	NR	NR	NR	NR	NR
Butanol	+	+*	NR	+	NR	+
*Electron acceptors with methanol*						
Sulphate	+	+	+	+	NR	+
Sulphur	−	−	+	+	NR	−
Sulphite	+	+	+	+	NR	+
Thiosulphate	+	+	+	+	NR	+
Fumarate	−	NR	NR	NR	NR	NR
Fe(III)	−	NR	NR	NR	NR	NR
Mn(IV)	−	NR	NR	NR	NR	NR
Nitrate	−	−	−	−	NR	−
DMSO	−	NR	NR	NR	NR	NR
*Fermentation*						
L-cysteine	−	NR	NR	NR	NR	NR
Lactate	+	+	+	−	+	NR
Glucose	−	NR	NR	NR	NR	NR
Glycerol	−	NR	NR	NR	NR	NR
Ethanol	(+)	NR	NR	NR	NR	NR
Methanol	(+)	NR	NR	NR	NR	NR
Pyruvate	+	+	+	+	+	+
Fumarate	+	+	NR	NR	NR	NR
Formate	(+)	NR	NR	NR	NR	NR
Benzoate	−	NR	NR	NR	NR	NR

None of the taxa were able to grow on sugars or amino acids. NaCl concentrations are additional to 0.3 g l^−1^ contained in basal medium. +  = positive, (+) = weak positive, − = negative, NR = not reported, * = from this study

**Fig. 2:**
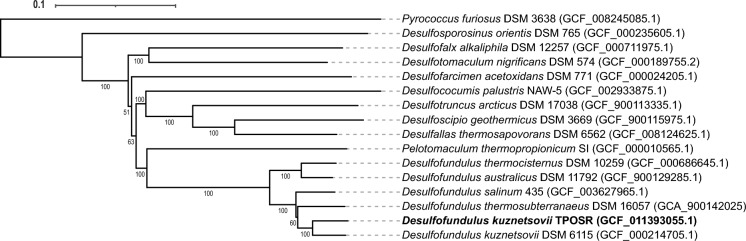
BLAST distance tree representing the phylogenomic relationship of strain TPOSR—DSM 110707 with the genus *Desulfofundulus* based on amino acid evolution. Type strains of the new clusters of the reclassified *Desulfofotomaculum* genus are included. *Pyrococcus furiosus* was included as outgroup. Reference accessions of genomes used are given in brackets. Tree reflects whole-proteome-based GBDP distances inferred with FASTME 2.1.6.1. Branch scale reflects expected number of substitutions per site and was scaled according to GBDP formula d5 and numbers below branches indicate pseudo-bootstrap support from 100 replications. The tree was rooted at the midpoint

**Fig. 3:**
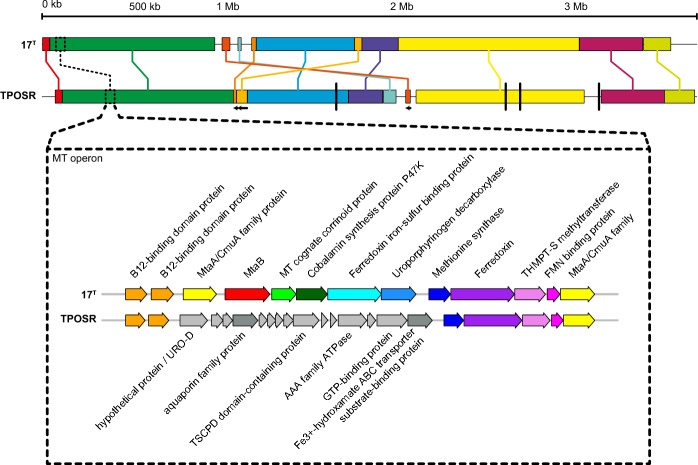
Depiction of genome homology of strain 17^T^ and TPOSR (top) and alignment of the MT operon of both strains (bottom). Top: Colinear homologous blocks are indicated with the same colour, inverted blocks of the TPOSR genome are represented by an arrow below, contigs of the TPOSR genome are divided by black vertical bars, the location of the MT operon is indicated with dashed boxes. Bottom: Arrows indicate protein coding sequences, homologous genes across both genomes share the same colour, grey arrows represent genes not found in both genomes, gene annotation is given or omitted if gene product is identified as hypothetical protein

The genome assembly of strain TPOSR resulted in 5 contigs with a total length of 3,760,898 bp and a G + C content of 55.2% mol mol^−1^. Of the 3792 annotated genes, 3579 were identified as protein coding genes. The structure of the genome shows strong resemblance to the genome of the type strain *D. kuznetsovii* strain 17^T^ with a total length of 3,601,386 bp and 3567 coding genes. Furthermore, both strains contain three copies of the 16S rRNA gene in their genome. Using the type strain genome server (Meier-Kolthoff and Göker 2019) and the genome BLAST distance phylogeny provided by DSMZ we were able to determine an ANI of 97.6% and dDDH (d^4^) of 80.6%, between strains TPOSR and 17^T^ with new species cutoff values of dDDH < 95% and ANI < 70%. These results confirm strain TPOSR belong to the species *Desulfofundulus kuznetsovii*. We generated a genome-based phylogenetic tree showing the close affiliation of strain TPOSR with the type strain 17^T^ and its position in the *Desulfofundulus* genus (Fig. 2). Genomic comparison of strain TPOSR with strain 17^T^ revealed a high degree of similarity in most investigated genes. Comparison of rRNA as well as of the sulphate reducer specific marker genes dissimilatory sulphite reductase (*dsrAB*) and adenosine-5′-phosphosulphate reductases (*apsA*) resulted in amino acid identity of 96% (*apsA*) up to 99% (*dsrA/B*). To detect rearrangements, deletions, or insertions in the genome of strain TPOSR, the alignment of the genome with the type strain was analysed using MAUVE (Darling et al. 2004) (Fig. 3). The alignment indicates large locally collinear blocks (LCBs) consisting of homologous regions without rearrangements. Most of the 17^T^ genome has homologous blocks in the TPOSR genome with minor structural rearrangements and sequence inversions between 1 and 2 Mb.

### Genomics of the methanol metabolism

We compared the methanol metabolism of *D. kuznetsovii* strains 17^T^ and TPOSR, identifying the MT system and/or ADH system. Previous proteomic analysis of strain 17^T^ revealed that this strain used both pathways for methanol conversion (Sousa et al. 2018). In a genomic context an MT operon was identified (AEG13700.1 to AEG13710.1) as well as an abundant ADH (AEG16455.1) and aldehyde ferredoxin oxidoreductase (AOR) (AEG16454.1). The MT operon of strain 17^T^ contains the subunits of the methyltransferase cluster MtaA (AEG13700.1 and AEG13710.1), MtaB (AEG13701.1) and MtaC (AEG13702.1) as well as accessory proteins involved in the biosynthesis of the required cofactor, vitamin B12. Interestingly the operon in strain 17^T^ shows some distinct differences to the operon present in strain TPOSR.

Genes at the upstream and downstream regions of the operon are present with very high sequence identity to its homologous gene in *D. kuznetsovii* strain 17^T^. This includes B12 related proteins, B12-binding domain proteins and methionine synthase, ferredoxin, THMPT S-methyltransferase, FMN binding protein and *mtaA/CmuA*. In contrast, several centrally located genes are absent in the TPOSR genome. The central part of the MT operon homolog in strain TPOSR contains several short open reading frames with most of them identified as hypothetical proteins. The longer sequences were annotated as an aquaporin family protein, a TSCPD domain protein, an AAA family ATPase, a GTP binding protein and a Fe^3+^-hydroxamate transporter substrate binding protein.

**Fig. 4:**
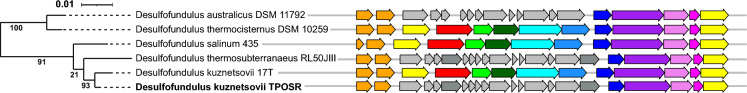
Methanol methyltransferase operon structures of *Desulfofundulus* taxa sorted by whole proteome phylogeny according to methods described in Fig. 2. Same colours represent same functional annotation. Individual annotation is given in Fig. 3

Using the operon structures of both strains of *D. kuznetsovii* as queries we were able to identify taxonomically close species with similar operon structures (Fig. 4). We identified the functional operon of strain 17^T^ in *Desulfofundulus salinum* 435 (95% nucleotide identity) and *Desulfofundulus thermocisternus* DSM10259 (92% nucleotide identity). Strikingly, *D. thermocisternus* was characterized as unable to grow with methanol (Nilsen et al. 1996) despite the presence of a complete MT system. The incomplete MT operon of strain TPOSR is found with high structural similarities in *D. thermosubterraneus* DSM16057 (95% nucleotide identity) and *D. australicus* DSM11792 (85% nucleotide identity). The former shares the complete set of inserted reading frames, while the latter shares the majority, but lacks the genes annotated as aquaporin family protein and Fe^3+^-hydroxamate transporter substrate binding protein. *D. australicus* can utilize methanol while *D. thermosubterraneus* was described not to metabolize it. The operon sequence similarity of species between the two structural groups ranges between 70 and 75%. The lack of functional annotation of the genes does not allow to hypothesize about possible functions of the inserted gene fragment.

The essential catalytically active methanol methyltransferase subunit B was not recognized in the MT operon or in any part of the remaining genome of strain TPOSR by either blast or hmmscan. Since MtaB catalyses the initial cleavage of the methyl group from methanol, we expect no MT activity in strain TPOSR. Growth of strain TPOSR on methanol therefore can be attributed to the activity of one of its alcohol dehydrogenases. In strain 17^T^ a 42 kDa alcohol dehydrogenase (AEG16455.1). It showed activity with methanol and ethanol (Visser et al. 2013). Additional ADH genes coding for similar sized proteins can be identified in the genome: AEG13809.1, AEG14230.1, AEG14235.1, AEG14239.1, AEG14856.1, AEG16458.1 and AEG16577.1. In strain TPOSR, 8 gene products could be annotated as alcohol dehydrogenases: NHM25678.1, NHM26105.1, NHM26109.1, NHM26114.1, NHM26139.1, NHM28481.1, NHM28580.1 and NHM28484.1. All sequences of both strains are annotated as iron-containing alcohol dehydrogenases and carry the typical Fe-atom binding site composed of three histidine and one aspartic acid residue (Larson et al. 2019). To assess similarities in the dehydrogenase genes of both strains, all sequences were oriented in the same direction and aligned using MAFFT version 7 (Katoh and Standley 2013). Pairwise amino acid sequence identity was determined using Clustal2.1 (Larkin et al. 2007).

Alignments indicate that most ADH genes found in *D. kuznetsovii* 17^T^, except AEG13809.1 match to a homologous gene in strain TPOSR with 94% to 100% identity (supplemental information, Figure S1). Local alignment of the unmatched sequence was carried out using BLAST. AEG13809.1 shares 50% amino acid identity with iron-containing alcohol dehydrogenases from *Desulfofundulus australicus*, *Desulfofundulus salinum* and strains from more distant genera. The genome of strain TPOSR also contains an ADH gene (NHM26139.1) without a highly identical homologue in strain 17^T^. This shares 97% amino acid identity with an iron-containing ADH of *Desulfofundulus thermobenzoicus* and 50% identity with ADHs from strains in the genera *Paucimonas, Clostridia* and *Deltaproteobacteria*. Most alcohol dehydrogenases in the genomes of both strains are near or adjacent to an AOR. Like ADH sequences also most AOR sequences are shared between both strains of *D. kuznetsovii* with high sequence identities.

### Proteome during growth on methanol

To confirm which enzymes are involved in the methanol metabolism of strain TPOSR, we performed proteomics analysis of strain TPOSR grown with methanol and lactate. To explore possible influence of cobalt availability on the abundance of proteins of both pathways we included analysis of cells grown on methanol without cobalt and vitamin B12. Growing *D. kuznetsovii* 17^T^ on methanol in the presence of B12 and cobalt, Sousa et al. (2018) observed abundance of a methanol methyltransferase operon as well as an alcohol dehydrogenase system. Cobalt starvation led to lower abundance of the MT system while the ADH system remained highly abundant.

Within the strain TPOSR MT operon structure, only a limited number of corresponding proteins were identified in the proteome data when cells were grown with both methanol and lactate. Specifically, two cobalamin-binding proteins originating from the genes located upstream of the insert (NHM26678.1 and NHM26677.1) were detected, albeit at low abundance. Notably, there was no significant differential expression observed in cells grown with methanol with and without cobalt. Similarly, the proteins located downstream of the insert (NHM26657.1 to NHM26661.1) were also present in the proteome. Among these, NHM26657.1 (MtaA/Cmua family protein), NHM26658.1 (pyridoxamine 5'-phosphate oxidase family), NHM26659.1 (tetrahydromethanopterin S-methyltransferase H), and NHM26660.1 (ASKHA domain-containing protein) exhibited a slight upregulation, just surpassing the significance cutoff (FDR = 0.05, s0 = 0.1). Regarding the inserted fragment in strain TPOSR, only NHM26675.1 was detected, with no significant differential expression observed. This finding suggests that the inserted fragment likely does not play a significant role in methanol metabolism. Furthermore, it is noteworthy that the proteins within the strain TPOSR operon homologous to those in the 17^T^ operon showed significantly less upregulation. This observation implies that these proteins are likely less crucial in the context of methanol metabolism in TPOSR. This is further supported by their lack of differential expression during growth, both in the presence and absence of cobalt. In strain 17^T^, it was observed that the upregulation of the MT operon during growth with methanol was inhibited by cobalt starvation. In summary, these findings collectively suggest that the MT operon in strain TPOSR is either not or to a much lower degree involved in methanol utilization. It is, therefore, expected that methanol utilization in strain TPOSR is primarily carried out through the ADH/AOR pathway.

**Fig. 5:**
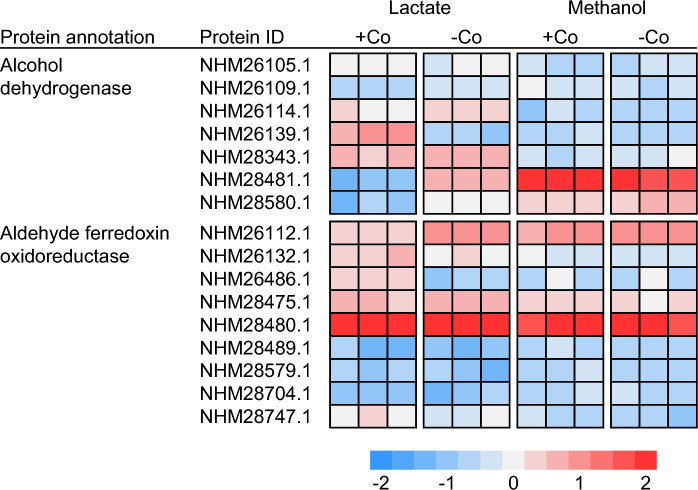
Abundance of alcohol dehydrogenases and aldehyde ferredoxin oxidoreductases of strain TPOSR during growth with lactate and methanol in medium with cobalt and vitamin B12 (+ Co) and absence thereof (− Co). Heatmap color codes represent z-score normalized log_10_ fold abundance of the respective protein as determined by label free quantification (LFQ) values

When strain TPOSR was grown with methanol, the expression of most genes in its methyl transferase homologue remained undetectable. However, several alcohol dehydrogenases and aldehyde ferredoxin oxidoreductases were detected (Fig. 5). Notably, the growth of *D. kuznetsovii* strain TPOSR with methanol led to a significant increase in the abundance of a 42 kDa alcohol dehydrogenase encoded by NHM28481.1 (fold change = 20,000). This alcohol dehydrogenase 99% amino acid identity with the highly abundant alcohol dehydrogenase observed in strain 17^T^ during growth with methanol. Additionally, we observed a slight upregulation (fold change = 46) of a second alcohol dehydrogenase (NHM28580.1), yet at a much lower level. The drastically higher fold change suggests the former (NHM28481.1) to be the primary ADH involved in the oxidation of methanol. Like the findings in strain 17^T^, the remaining alcohol dehydrogenases did not exhibit differential expression suggesting they are not involved in methanol degradation. In the proteome, we also detected a highly abundant aldehyde ferredoxin oxidoreductase, NHM28480.1 (log10 LFQ = 9.8). The gene encoding this enzyme is located immediately upstream of the highly abundant alcohol dehydrogenase gene (NHM28481.1) and shares 99% amino acid identity with its counterpart in strain 17^T^. These findings strongly suggest that this specific alcohol dehydrogenase/aldehyde ferredoxin oxidoreductase pair is primarily responsible for the growth of strain TPOSR with methanol. However, further research is needed to determine whether the other alcohol dehydrogenases in the TPOSR genome serve redundancy purposes or participate in the metabolism of other substrates.

Sousa et al. (2018) demonstrated the advantage of a two-pathway system, like that found in strain 17^T^, in cobalt-limited deep-subsurface environments. Expanding on this, this research highlighted the presence of an incomplete MT operon in strain TPOSR and various other isolates. These findings suggest the existence of a distinct ecological niche where the cobalt independent ADH pathway renders the additional MT pathway unnecessary. This adaptation might be linked to more severe cobalt scarcity, resulting in predominantly inactive MT pathways. Remarkably, a purely ADH dependent methanol metabolism pathway, as commonly used by aerobic methanol degraders, facilitates utilization of methanol of this group of anaerobic sulphate reducers.

### Growth of strains 17^T^ and TPOSR with and without cobalt

**Fig. 6:**
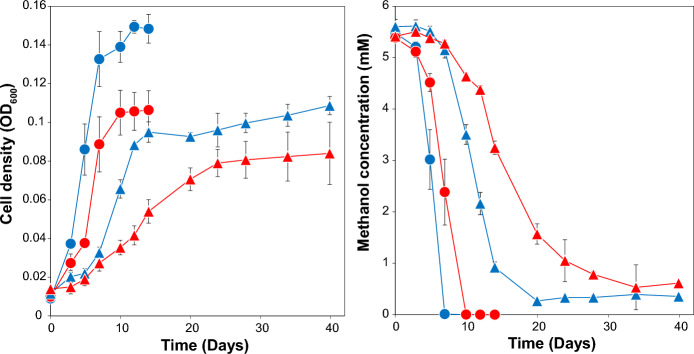
Growth (**A**) and medium methanol concentration (**B**) of strain TPOSR (blue lines) and strain 17^T^ (red lines) with methanol and sulphate (20 mM) in presence (circles) and absence (triangles) of cobalt and B12. Lines represent averages of triplicates. Error bars indicate standard deviation of triplicate measurements. OD_600_ and methanol measurements for cultures grown with cobalt were completed and terminated at day 14

The presence of two distinct pathways was hypothesized to be advantageous in metabolic competition in the methanol and cobalt limiting deep sub-surface habitat of the strains. The alcohol dehydrogenase pathway is expected to offer an alternative methanol assimilation pathway during cobalt limitation. We conducted growth studies of *D. kuznetsovii* strains 17^T^ and TPOSR in presence and absence of cobalt to investigate possible effects of the absence of MtaB in strain TPOSR. Both strains were inoculated into bottles with medium containing methanol and sulphate—one set of bottles with vitamin B12 and cobalt and one without. In regular intervals OD_600_ was measured as well as medium methanol concentration (Fig. 6). When grown with cobalt both strains grow without lag phase to final cell densities of OD_600_ 0.15 and 0.11 for strain TPOSR and 17^T^ respectively. The reason for the difference in biomass yield is yet unclear, but the expenditure of energy for the expression of the MT system in strain 17^T^ may contribute to this.

In absence of cobalt, both strains exhibit an extended lag phase of 3 days and final cell density is reduced by about 30% from 0.15 and 0.11 to 0.1 and 0.08 for strain TPOSR and 17^T^ respectively. Interestingly, maximum growth rate of strain TPOSR was reduced by 57% (from 0.073 to 0.032 day^−1^) while maximum growth rate of strain 17^T^ was reduced by 71% (0.059–0.017 day^−1^). In the presence of cobalt both strains rapidly consume all available methanol within 7–10 days. Cells grown in absence of cobalt show reduced methanol uptake rates in line with the reduced growth rates. Interestingly, for both strains residual methanol in the medium was detected even after 40 days of incubation. Residual methanol concentration in bottles with cells of 17^T^ showed consistently higher amounts of methanol (0.613 ± 0.03 mM and 0.355 ± 0.025 mM in 17^T^ and TPSOR, respectively). Besides methanol no other products were detected during this time. The apparent increase in OD_600_ and the increase in measurement variation after the stable methanol concentration was reached (day 20 and beyond) can be attributed to small amounts of precipitation that occur after extended incubation of the medium at high temperature.

Results indicate an inhibitory effect of cobalt starvation on the growth and methanol uptake of both strain 17^T^ and strain TPOSR. This inhibition is especially pronounced in cultures of strain 17^T^. This is likely caused by the lack of activity of the methanol methyltransferase system in strain 17^T^ at cobalt starvation. Previous research suggests that at higher methanol concentrations the ADH system is employed while the MT system is operating predominantly at lower methanol concentrations (Sousa et al. 2018). In line with this, the growth and methanol consumption are inhibited due to missing MT activity caused by cobalamin deficiency. The impairment of strain 17^T^ seemingly increases with reducing methanol concentrations reflecting its importance at low methanol concentrations. Similar growth and methanol uptake rates in early stages of the culture suggest similar performance of both ADH systems as suggested by the high homology of ADH/AOR proteins. The lack of MT activity in strain 17^T^ can explain the incomplete methanol uptake in cobalt starved conditions. The presence of residual methanol in bottles with strain TPOSR and its impaired growth remains unexplained since the employed ADH is independent of cobalt availability. A possible explanation is the inhibition of other cobalt or vitamin B12 dependent enzymes that can affect growth of the strain. An example of this is the cobalt dependant methionine synthase (EC:2.1.1.13). It facilitates the interconversion of homocysteine and methyl-tetrahydrofolate to methionine and tetrahydrofolate and is thereby integral for the acetyl-CoA pathway. Lack of the required cofactor could therefore be imagined to have an impact on the cells growth. Alternatively, it has been demonstrated that cobalt availability modulates substrate affinity of some ADHs (Vanni et al. 2000) potentially explaining the remaining amount of methanol, but this remains to be investigated for now.

## Conclusion

Through genomic analysis, we confirmed strain TPOSR affiliates with *Desulfofundulus kuznetsovii*, being closely related to strain 17^T^. The physiological similarity and high ANI values underscore their close phylogenetic affiliation within the *Desulfofundulus* genus. Analysis of the methanol metabolism pathways in both strains revealed that strain TPOSR lacks the crucial *mtaB* gene in its MT pathway, which has been shown to be important for methanol metabolism of strain 17^T^ with cobalt. This suggests that strain TPOSR relies on one of its alcohol dehydrogenases for methanol assimilation. Similar MT operon structures in related strains suggest this is a more widespread form of anaerobic methanol metabolism. Proteomics analysis indicated the involvement of a specific ADH and AOR in the metabolism of methanol that are highly identical to the ones abundant in 17^T^ growing with methanol. No significant abundance of MT associated proteins in the TPOSR proteome confirms its reliance on ADH activity. Physiological studies demonstrate that methanol utilization of both strains is impaired when grown without cobalt. This effect is more pronounced in strain 17^T^ likely due to missing MT activation caused by cobalamin deficiency. In essence, this research sheds first light on a yet undescribed form of methanol metabolism of anaerobic sulphate reducing organisms.

## Supplementary Information

Below is the link to the electronic supplementary material.

Supplementary file1 (XLSX 30 kb)

Supplementary file2 (DOCX 1073 kb)
