# Supplementary material for: Alcohol dehydrogenase system acts as the sole pathway for methanol oxidation in Desulfofundulus kuznetsovii strain TPOSR
Source: Antonie Van Leeuwenhoek. 2024 Mar 1;117(1):47. doi: 10.1007/s10482-024-01937-1 (PMC10907483; doi:10.1007/s10482-024-01937-1)
Supplement: Supplementary file 2 — Supplementary file2 (DOCX 1073 kb) [file 10482_2024_1937_MOESM2_ESM.docx]

Supplemental information


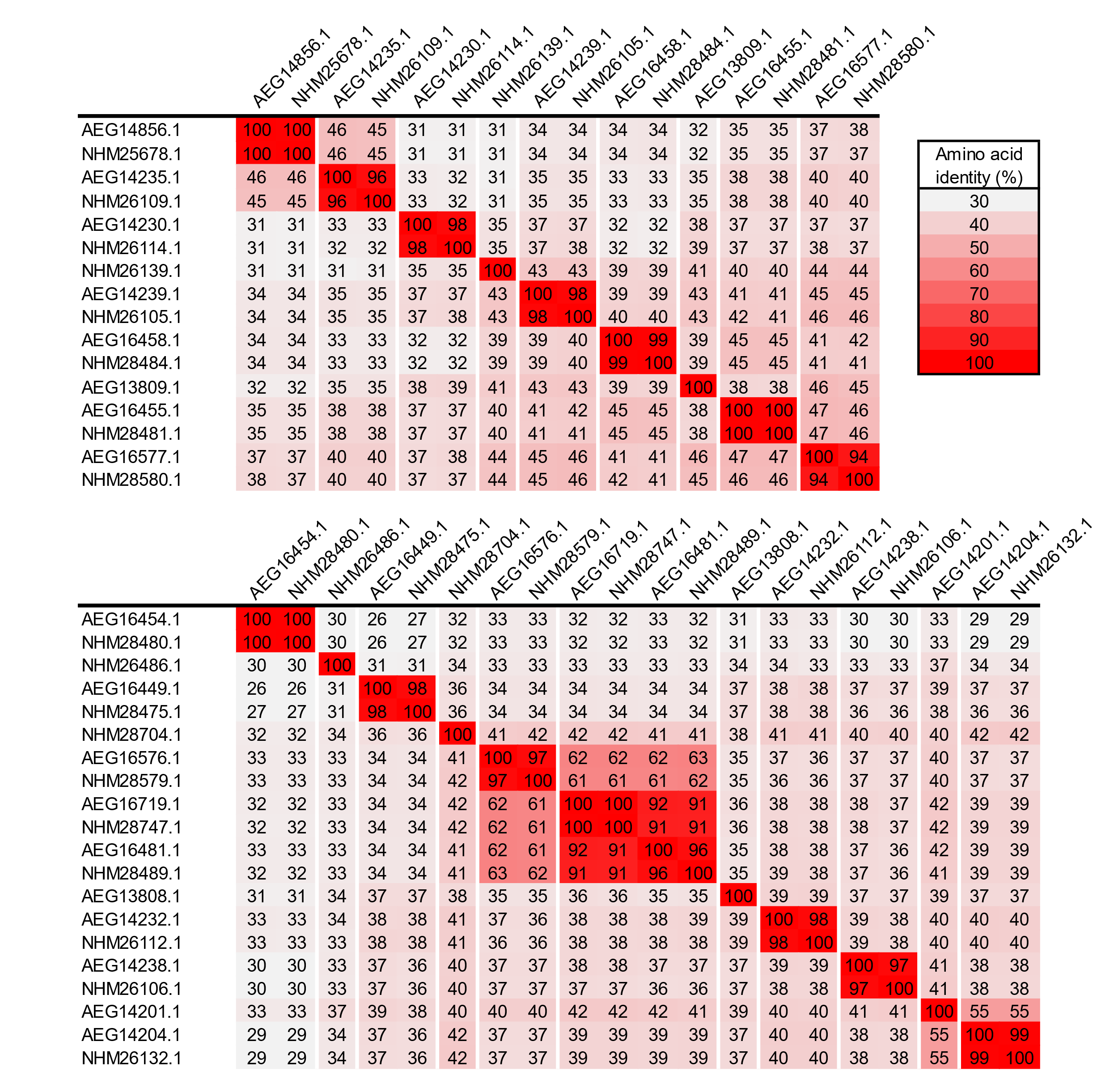


**Online Resource 1** Pairwise amino acid alignment of Alcohol dehydrogenase (A) and Aldehyde ferredoxin oxidoreductase (B) sequences from strain 17^T^ (AEG) and strain TPOSR (NHM). Numbers indicate the percentage of amino acid sequence identity between pairwise alignments.
